# Role of MMP-2 (-1306 C/T) Polymorphism in Pituitary Adenoma

**DOI:** 10.1155/2016/2839697

**Published:** 2016-03-09

**Authors:** Brigita Glebauskiene, Rasa Liutkeviciene, Alvita Vilkeviciute, Loresa Kriauciuniene, Giedrimantas Bernotas, Arimantas Tamasauskas, Dalia Zaliuniene

**Affiliations:** ^1^Medical Academy, Department of Ophthalmology, Lithuanian University of Health Sciences, Eiveniu 2, 50009 Kaunas, Lithuania; ^2^Neuroscience Institute, Medical Academy, Lithuanian University of Health Sciences, Eiveniu 2, 50009 Kaunas, Lithuania; ^3^Medical Academy, Department of Neurosurgery, Lithuanian University of Health Sciences, Eiveniu 2, 50009 Kaunas, Lithuania

## Abstract

*Purpose*. To determine if the frequency of the genotype of* MMP-2 (-1306 C/T) Rs243865 *has an influence on the development of pituitary adenoma (PA).* Methods*. The study enrolled *n* = 84 patients with PA and a random sample of the population *n* = 318 (reference group). The genotyping test of* MMP-2 (-1306 C/T) *was carried out using the real-time polymerase chain reaction method.* Results*. Analysis of* MMP-2 (-1306 C/T) *gene polymorphism has not revealed any differences in the genotype (*C/C, C/T,* and* T/T*) distribution between the PA patients and the reference group (as follows: 50%, 44%, and 6% versus 59.75%, 33.96%, and 6.29%).* MMP-2 (-1306) C/C* genotype was rarely observed in noninvasive PA compared to healthy controls: 35.1% versus 59.75%; *p* = 0.0049, as well C/C genotype being more frequently detected in nonrecurrence PA compared to healthy controls: 46.5% versus 59.75%; *p* = 0.0468.* MMP-2 (-1306) C/T* genotype was more frequently present in PA females compared to healthy controls females: 49.1% versus 33.66%; *p* = 0.041.* Conclusion*. Patients with noninvasive and nonrecurrence pituitary adenoma were the carriers of the C/C genotype significantly more frequently than their control counterparts and the C/T genotype in females was more frequent.

## 1. Introduction

Pituitary adenoma (PA) is a common benign monoclonal neoplasm accounting for approximately 15% to 20% of primary intracranial tumours [1]. Ezzat et al. [2] reported the estimated prevalence rates of pituitary adenomas to be 14.4% to 22.5% in pooled autopsy and radiological series, respectively. The pituitary gland is localized in a dural bag attached to the inferior aspect of the diaphragm of the sella and surrounded by venous spaces that correspond laterally to the cavernous sinuses [3]. PA may grow large and extend into the surrounding structures resulting in neurological complications including visual impairment. 6% to 10% of pituitary adenomas involve the cavernous sinus [4–9]. PA is a disease of multifactorial etiology, the occurrence of which is influenced by alterations in hormonal regulation and hormone receptors, dysregulated growth factors and alterations in their receptors, abnormalities in signaling proteins that transduce the signals of these stimuli, and changes in cell-cycle regulators. In addition, the neoplastic process is associated with altered cell-stromal interactions that have a role in the morphogenesis of pituitary tumours [10]. Recently, great attention in the PA pathogenesis has been drawn to the search of new epigenetic and genetic factors. To invade, tumour cells must undergo several changes in molecular pathways in accordance with invasion-associated cellular activities, namely, cell-cell adhesion, cell-matrix adhesion and ectopic survival, migration, and proteolysis [11]. Matrix metalloproteinases (MMPs), a family of zinc-dependent endopeptidases, also called matrixins, play an important role in the process of degradation of the extracellular matrix (ECM) and basement membrane (BM) in relation to tumour invasiveness, metastasis, and angiogenesis [12–18]. Many factors might induce MMPs production: cytokines, growth factors, physical stress, cell-extracellular matrix, and cell-cell interaction [19].

MMP-2 is a member of the MMP family and is capable of hydrolyzing type IV collagen, which is the main component of the BM [13, 15]. Several studies have shown that MMP-2 plays an important role not only in tumour invasion and metastasis, but also in cancer development [20, 21].

Numerous studies have shown that MMP-2 is overexpressed in various human tumours, including breast cancer [22–27], lung cancer [28], colorectal tumours [29], pancreatic carcinoma [30], and gastric and esophageal cancers [31–35]. Some studies have also showed the expression of MMP-2 in human gliomas [36–38]. There are not many studies analyzing MMP-2 expression in PA [5, 39, 40] and to the best of our knowledge there are no studies that have examined the* MMP2* -1306 C/T polymorphism in patients with PA.

*MMP *polymorphisms can be caused by nucleotide changes within the promoter region by insertions, substitutions, or microsatellite instability [41]. Price et al. [42] reported a single nucleotide polymorphism in the promoter of the* MMP2* gene (-1306 C/T). -1306 C→T transition is located in a core recognition sequence of Sp1 (CCACC box), which abolishes the Sp1-binding site and consequently diminishes promoter activity. Another C to T transition located at nucleotide -735 in the promoter region of* MMP-2* has been identified [43].

Numerous studies have been carried out to look for the possible association between the MMP2 -1306 C>T polymorphism and risk of human cancers (colorectal, breast, gastric, esophageal, prostate, lung, and oral cancer) (reviewed in [44, 45]).

To our knowledge, no studies have investigated the association between the* MMP-2 (-1306 C/T)* gene polymorphism and PA development. Therefore, the aim of this study was to determine the association between the* MMP-2 (-1306 C/T)* gene polymorphism and the development of PA.

## 2. Materials and Methods

Permission (Number P2-9/2003) to undertake the study was obtained from the Kaunas Regional Biomedical Research Ethics Committee. The study was conducted in the Departments of Ophthalmology and Neurosurgery, Lithuanian Health Sciences University Hospital.

Study participants comprised 84 subjects with a diagnosis of pituitary adenoma and 318 persons from the reference group.

*Reference Group Formation*. The reference group involved 318 subjects according to their age and gender, considering the pituitary adenoma group structure. It was constructed from the following:

1. A random sample of the Kaunas population aged 45–74 years collected within the international HAPPIE (Health, Alcohol and Psychosocial Factors in Eastern Europe) project (1) by the Laboratory of Population Research at the Institute of Cardiology of the Lithuanian University of Health Sciences (LUHS).
2. A random sample of the Lithuanian population aged 25–65 years collected within the international CINDI (Countrywide Integrated Non-Communicable Disease Intervention) project (2) by the Laboratory of Preventive Medicine at the Institute for Biomedical Research of the LUHS.
3. A random sample of the Kaunas population older than 65 years collected within the “Kaunas Healthy Ageing Study” by the Geriatric Clinic and Laboratory of Molecular Cardiology, Institute of Cardiology of the LUHS (3).

The reference group was created by taking into consideration the distribution of age and gender in the pituitary adenoma group. Therefore, the medians of the patient age of the reference group and the pituitary group did not differ statistically significantly (*p* < 0.05).

Demographic data of the study subjects are presented in Table 1.

The inclusion criteria were as follows: (1) determined and confirmed PA via MRI; (2) patient's general good condition; (3) patient's consent to take part in the study; (4) age ≥ 18 years, (5) no other brain or other localization tumours.

### 2.1. Radiological Evaluation

All pituitary adenomas were analyzed based on MR imaging findings. The suprasellar extension and sphenoid sinus invasion by PAs were classified according to Hardy classification, modified by Wilson [46]. The degree of suprasellar and parasellar extension was graded as stages A–E. The degree of sellar floor erosion was graded as grades I–IV. Grades I-II mean that sellar floor is intact and was considered as noninvasive PA, grade III shows localized sellar perforation, and grade IV shows diffuse destruction of sellar floor which is the sign of invasive PA. Knosp classification system was used to quantify the invasion of the cavernous sinus. Grade 0: no involvement of cavernous sinus represents the normal condition; grades 1 and 2: the tumour pushes into the medial wall of the cavernous sinus but does not go beyond a hypothetical line extending between the centres of the two segments of the internal carotid artery (grade 1) or it goes beyond such a line, but without passing a line tangent to the lateral margins of the artery itself (grade 2); grade 3: the tumour extends laterally to the internal carotid artery within the cavernous sinus; grade 4: total encasement of the intracavernous carotid artery [47]. According to Knosp classification, only grades 3 and 4 pituitary tumours were considered to be invasive.

### 2.2. DNA Extraction and Genotyping

The DNA extraction and analysis of the gene polymorphism of MMPs were carried out at the Laboratory of Molecular Cardiology at the Institute of Cardiology of the LUHS for control group and at the Laboratory of Ophthalmology at the Institute of Neuroscience of the LUHS for the PA patient group. The DNA was extracted from the venous blood of patients using the Genomic DNA Purification Kit (Thermo Fisher Scientific) according to the recommendations of the manufacturer or the silica gel column method utilizing the genomic DNA extraction kit SorpoClean*™* Genomic DNA Extraction Module (SORPO Diagnostics) according to the recommendations of the manufacturer.

The genotyping test of MMP-2 (-1306 C/T) was carried out using the real-time polymerase chain reaction (PCR) method. Applied Biosystem (USA) kits were used for the genotyping of MMP-2 (-1306 C/T) (rs243865). To ensure internal control, 20 samples were sequenced at the Sequencing Center of the Institute of Biotechnology, and the received results confirmed the reiteration and precision of the data. The genotyping was performed using the HT 7900 real-time PCR quantification system (Applied Biosystems, USA). The real-time PCR reagents (2x Maxima*™* Probe/ROX qPCR Master mix buffer, fluorescent dye labeled markers, sterile ddH2O) were taken out from an environment of –20°C and were thawed at room temperature. The thawed reagents were centrifuged (10,000 rpm) and stored in an ice tub. An appropriate real-time PCR mixture of MMP-2 (-1306 C/T) was prepared for determining single nucleotide polymorphism (SNP).

9 *μ*L of the PCR reaction mixture was poured into each well of the microtiter plate with 96 wells and then 1 *μ*L of matrix DNA of the samples (~10 ng) and 1 *μ*L of negative control (−K) were added. An optic film was pasted on the microtiter with 96 wells and the microtiter was centrifuged for 15 seconds at 10,000 rpm.

During the genotyping the following real-time PCR programs were used: Allelic Discrimination and Absolute quantification. Then, the work was continued following the manual provided by the manufacturer (http://www.appliedbiosystems.com/ Allelic Discrimination Getting Started Guide). After that, the Allelic Discrimination program was completed, the genotyping results were received. The program determined the individual genotypes according to the fluorescence intensity rate of different detectors. A molecular marker labeled with VIC fluorescent dye or Yakima Yellow was chosen for the *x* axis and a molecular marker labeled with FAM fluorescent dye was selected for the *y*-axis.

### 2.3. Statistical Analysis

Statistical analysis was performed using the SPSS/W 20.0 software (Statistical Package for the Social Sciences for Windows, Inc., Chicago, IL, USA). The data are presented as minimum, maximum, and median. The frequencies of genotypes (in percentage) are presented in Table 2. Hardy-Weinberg analysis was performed to compare the observed and expected MMP genotype frequencies using the *χ* ^2^ test for all groups. The distribution of the MMPs (SNP) in the PA and control groups was compared using the *χ* ^2^ test or Fisher exact test. Binomial logistic regression analysis was performed to estimate the impact of genotypes on PA development. Odds ratios (OR) and 95% confidence intervals are presented. The selection of the best genetic model was based on the Akaike Information Criterion; therefore, the best genetic models were those with the lowest Akaike Information Criterion values. Differences were considered statistically significant when *p* < 0.05.

## 3. Results

The genotyping of* MMP-2 (-1306) C/T* was performed in patients with PA and in the control group subjects (Table 2). The distribution of the analyzed MMP genotypes and allele frequencies in patients with PA and in the control group matched the Hardy-Weinberg equilibrium.* MMP-2 (-1306) C/T* gene polymorphism analysis in the overall group has not revealed any differences in the genotypes distribution between patients with PA and control group patients (Table 2).

*MMP-2 (-1306) C/T *gene polymorphism analysis in males and females with PA has not revealed any statistical significant differences in the genotype (*C/C, C/T* and* T/T*) distribution (as follows: 45.5%, 49.1%, and 5.5% versus 58.6%, 34.5%, and 6.9%) (Table 3). When comparing* MMP-2* genotype distribution in healthy females and females with PA we have revealed significant differences.* MMP-2 (-1306) C/T* genotype was more frequently present in PA females compared to healthy controls females: 49.1% versus 33.66%; *p* = 0.041.* MMP-2 (-1306)* C/C and C/T genotypes have not revealed any statistically significant differences when healthy females and females with PA were compared:* MMP-2 (-1306)* C/C genotype 59.02% versus 45.5%; *p* = 0.09; and T/T genotype 15% versus 3%, *p* = 0.772.

Binomial logistic regression analysis in the patients with PA and in the control group was performed (Table 4). This analysis revealed that there were no statistically significant variables in the models of the patients with PA and in the control group.

Binomial logistic regression analysis in the patients with PA and in the control group by gender was performed (Table 4). There were no statistically significant variables in the models of the pituitary adenoma and control groups. Binomial logistic regression analysis in the patients with PA and in the control group by gender was performed as well (Table 5). There were no statistically significant variables in the models of males. In females this analysis revealed that the codominant (*p* value = 0.043) and overdominant (*p* value = 0.037) variables were statistically significant.

*MMP-2 (-1306) C/C* genotype was rarely observed in noninvasive PA compared to healthy controls: 35.1% versus 59.75%; *p* = 0.0049, as well C/C genotype being more frequently detected in nonrecurrence PA compared to healthy controls: 46.5% versus 59.75%; *p* = 0.0468 (Tables 6 and 7). These results could be explained by increased expression of* C/C* genotype.

Binomial logistic regression analysis in noninvasive PA and in the control group was performed (Table 8). In noninvasive PA group this analysis revealed that the codominant (*p* value = 0.003), dominant (*p* value = 0.005), overdominant (*p* value = 0.003), and additive (*p* value = 0.028) variables were statistically significant. Binomial logistic regression analysis in the patients with nonrecurrence PA and in the control group was performed as well (Table 8). In nonrecurrence PA group this analysis revealed that the codominant (*p* value = 0.039), dominant (*p* value = 0.042), and overdominant (*p* value = 0.049) variables were statistically significant.

## 4. Discussion

Pituitary tumours are benign but do not uncommonly invade locally into adjacent tissues such as the cavernous sinus and dura. Early prediction of which pituitary tumours will recur and/or exhibit an invasive phenotype remains difficult despite the introduction of several tissue-based molecular markers [48].

The importance of* MMP-2 (-1306)* gene polymorphism in the susceptibility of various tumours has been shown in numerous studies [47–50]. In addition, MMP-2 has been shown to be overexpressed in PA [5, 39, 40]. On the basis of these findings, we sought to examine whether the polymorphism in the* MMP2 (-1306)* promoter could have an impact on the risk of PA development. We analyzed 84 PA patients and 318 age- and sex-matched controls for the -1306 C/T polymorphism in the* MMP-2* promoter. Our results demonstrated that* MMP-2 (-1306 C/T)* gene polymorphism has not revealed any differences in the genotype (*C/C, C/T,* and* T/T*) distribution between the PA patients and the reference group (as follows: 50%, 44%, and 6% versus 59.75%, 33.96%, and 6.29%), but* MMP-2 (-1306) C/T* genotype was more frequently present in PA females compared to healthy controls females: 49.1% versus 33.66%; *p* = 0.041.

To our knowledge, there are no studies which have explored the relationship between the polymorphisms in* MMP2* -1306 C/T and the development of PA. However, several studies have analyzed MMP-2 expression in PA [5, 39, 40]. Liu et al. [39] have found that the MMP-2 score of PAs with cavernous sinus invasion (3.9 ± 0.5) was significantly higher than those without invasion (2.3 ± 0.2; *p* < 0.01). There was no difference in the MMP-2 score between macroadenomas (3.0 ± 0.3) and microadenomas (2.1 ± 0.4; *p* > 0.05), and also no difference between the functioning adenomas (2.8 ± 0.3) and nonfunctioning adenomas (2.8 ± 0.3; *p* > 0.05) [5]. MMP-2 mRNA expression was also intense in invasive pituitary adenomas and was significantly higher in invasive pituitary adenomas than those without invasion (68.2 ± 15.3; 21.8 ± 8.2; *p* < 0.05). Pereda et al. [40] have observed the activities of MMP-2 and MMP-9 together with the expression of membrane-type MMP and tissue inhibitor of metalloproteinase-1 in all types of human pituitary adenomas. They found high levels of MMP activity and low levels of tissue inhibitor of metalloproteinases, indicating a high level of extracellular matrix-degrading activity in PAs.

Numerous studies have demonstrated overexpressed MMP-2 in various tumours: breast cancer [22–27], lung cancer [28], colorectal tumours [29], gastric and esophageal cancers [31–35], and pancreatic carcinoma [30]. Some studies have reported the expression of MMP-2 in human gliomas [36–38].

Numerous studies have also been carried out to look for an association between the MMP-2 -1306 C/T polymorphism and risk of other human tumours, but the results remain controversial [49–61].

Yu et al. [49] in their research have found that the allele frequency of* MMP2* -1306 C was significantly higher among cases of lung cancer than among controls (0.91 versus 0.83). Subjects with the* CC* genotype had an overall 2-fold increased risk of developing lung cancer [adjusted OR 2.18; 95% confidence interval (CI), 1.70–2.79] compared with those with the* CT* or* TT* genotype. In another study Yu et al. [55] have reported that the C_−1306_–C_−735_ haplotype in the* MMP-2* promoter contributes to risk of the occurrence and metastasis of esophageal squamous cell carcinoma by increasing the expression of MMP-2. In another study this group of researches has found that subjects with the* CC* genotype had a more than 3-fold increased risk [adjusted OR 3.36, 95% confidence interval 2.34–4.97] for developing gastric cardia adenocarcinoma compared with those with the variant* CT* or* TT* genotype. The increased risk was found to be more pronounced in smokers and younger subjects. No significant association was demonstrated between the* MMP2* polymorphism and the risk of metastasis of the cancer at the time of diagnosis, with the OR being 0.90 (95% confidence interval 0.36–2.20) for the* CC* genotype [50]. In breast cancer research Miao et al. [50] have found that the variant* MMP2* genotype (-1306 CT or TT) was associated with substantially reduced risk of breast cancer [OR 0.46; 95% confidence interval (95% CI), 0.34–0.63], compared with the CC genotype. Grieu et al. [62] have also reported that MMP-2 TT homozygous patients had smaller breast tumours (*p* = 0.006) and contained lower concentrations of the estrogen receptor (ER; *p* = 0.002) compared to patients with the MMP-2 CC or CT genotype. Homozygosity for the MMP-2 -1306 T allele was associated with markedly different patient survival depending upon tumour ER status. For patients with ER negative tumours, the MMP-2 TT genotype was associated with poor survival (2/8 patients alive at end of study, 25%) compared to the CC or CT genotypes (59/70, 84%; *p* < 0.001). For patients with ER positive tumours, the MMP-2 TT genotype was associated with a trend for very good survival (10/10, 100%) compared to the CC or CT genotypes (130/157, 83%; *p* = 0.16).

Lin et al. in their study [52] provided evidence that -1306* C→T* polymorphism in the MMP-2 promoter is a susceptibility factor for the development of oral squamous cell carcinoma, with the* CC* genotype being associated with the increase of risk. O-charoenrat and Khantapura [58] have reported that subjects with the MMP2 CC genotype were associated with a significantly increased risk [adjusted OR 1.97; 95% confidence interval (95% CI), 1.23–3.15] for developing HNSCC compared with those with the variant genotype (-1306 CT or TT).

Xu et al. [60] have found that the frequency of MMP-2 CC genotype was significantly higher in colorectal cancer patients when compared with controls (OR, 1.959; 95% CI, 1.055–3.637). Srivastava et al. [61] have found that patients with MMP2 (-1306) CT genotype as well as T allele were at higher risk of prostate cancer (*p* = 0.018; OR = 1.68 and *p* = 0.015; OR = 1.52). This effect was even more evident in the case of the T allele carrier (CT + TT) (*p* = 0.011; OR = 1.71). Shao et al. [63] have reported that the risk of nasopharyngeal carcinoma was significantly increased in young (<60 years) subjects with the -1306 CC genotype (OR = 1.52, 95% CI = 1.01–2.29). Wieczorek et al. [56] have found that the combined genotype MMP2 -1306 C/T (rs243865) allele T with MMP9 -1562 C/T (rs3918242) allele T increased bladder cancer risk (OR 2.00, 95% CI 1.10–3.62; *p* = 0.022).

On the basis of these findings, we hypothesized that the -1306 C/T polymorphism in* MMP-2* might also have impact on individual susceptibility to PA.

Some authors have not found an association of MMP-2 (-1306 C/T) polymorphism with tumours. Rollin et al. [53] have found no difference in -1306 C/T MMP-2, -735 C/T MMP-2, and -1562 C/T MMP-9 genotypes between cases of non-small cell lung cancer and controls. Eftekhary et al. [54] have found no statistically significant differences in genotype and allele frequencies of MMP-2 (-1306 C/T) between patients with esophageal squamous cell carcinoma and controls (*p* > 0.05). A significant association of the MMP-2 (-1306 C/T) polymorphism with GBM (*p* = 0.475) was not found by Kumar et al. [59] suggesting that MMP-2 (-1306 C/T) polymorphism is not associated with increased GBM susceptibility. Kawal et al. [57] have not found a significant association of MMP-2 (-1306 C/T) polymorphism with oligodendroglioma (*p* = 0.54).

To the best of our knowledge, this is the first study to examine the relationship between the* MMP-2* (-1306 C/T) polymorphism and PA risk. Our study showed a significantly greater prevalence of the C/T genotype in females than their control counterparts. Further studies with a larger number of patients, however, are necessary in order to better understand the real value of such a normative database in developing of PA.

Our study suggests that the effects of polymorphisms of* MMPs* on PA risk deserve further investigation.

## Figures and Tables

**Table 1:** Demographic characteristics of patients with pituitary adenoma (PA) and reference group subjects.

Group	*N*	Age, year (min./max. median)	Males, *n* (%)
PA	84	19/87/52.5	29 (34.5)
Reference	318	25/87/51	113 (35.5)
*p* value	—	0.88	0.86

**Table 2:** Frequency of *MMP-2 (-1306 C/T)* genotype in the patients with pituitary adenoma (PA) and in the control group.

Gene marker	Genotype/allele	Frequency (%)				
		Control group *n* (%) (*n* = 318)	*p* HWE	PA group *n* (%) (*n* = 84)	*p* HWE	*p* value
*MMP-2 (-1306) Rs243865*	Genotype		0.383		0.390	
	C/C	190 (59.75)		42 (50.00)		*χ* ^2^ = 2.980
	C/T	108 (33.96)		37 (44.00)		*p* = 0.225
	T/T	20 (6.29)		5 (6.00)		
	Total	318 (100)		84 (100)		
	Allele					
	C	0.767		0.720		
	T	0.233		0.280		

MMP: matrix metalloproteinase; *p* value: significance level (alfa = 0.05); *p*-value HWE: significance level (alfa = 0.05) by Hardy-Weinberg equilibrium.

**Table 3:** Frequency of *MMP-2 (-1306 C/T)* genotype in the patients with pituitary adenoma (PA) and in the control group by gender.

Gene marker	Genotype/allele	Frequency (%)							
		Control group		*p* HWE	*p* value	PA group		*p* HWE	*p* value
		*n* (%)				*n* (%)			
		Females	Males			Females	Males		
		*N* = 205	*N* = 113			*N* = 55	*N* = 29		
*MMP-2* *(-1306)* *Rs243865*	Genotype			0.488				0.394	
	C/C	121 (59.02)	69 (61.06)		0.811	25 (45.5)	17 (58.6)		0.359
	C/T	**69 **(33.66)^*∗*^	39 (34.51)		0.803	**27 **(49.1)^*∗*^	10 (34.5)		0.251
	T/T	15 (7.32)	5 (4.42)		0.347	3 (5.5)	2 (6.9)		1.0
	Total	205 (100)	113 (100)			55 (100)	29 (100)		
	Allele								
	C	311 (75.85)	177 (78.32)			77 (70)	44 (75.86)		
	T	99 (24.15)	49 (21.68)			33 (30)	14 (24.14)		

^*∗*^ *p* = 0.0412.

MMP: matrix metalloproteinase; *p* value: significance level (alfa = 0.05); *p*-value HWE: significance level (alfa = 0.05) by Hardy-Weinberg equilibrium.

**Table 4:** Binomial logistic regression analysis in the patients with pituitary adenoma (PA) and in the control group.

Model	Genotype	OR (CI 95%)	*p* value	AIC
Codominant	T/T	1		415.177
	T/C	0.645 (0.391–1.065)	0.087	
	C/C	0.884 (0.314–2.490)	0.816	

Dominant	CC	1		413.540
	T/C + T/T	0.647 (0.416–1.092)	0.109	

Recessive	C/C + C/T	1		416.093
	T/T	1.060 (0.386–2.914)	0.909	

Overdominant	T/T + C/C	1		413.231
	C/T	0.653 (0.400–1.066)	0.088	

Additive	T allele	0.784 (0.535–1.148)	0.211	414.568

**Table 5:** Binomial logistic regression analysis in pituitary adenoma (PA) and the control women by gender.

Model	Genotype	OR (CI 95%)	*p* value	AIC
Males				
Codominant	CC	1		149.476
	CT	0.961 (0.401–2.303)	0.929	
	TT	0.616 (0.110–3.452)	0.582	
Dominant	CC	1		147.705
	T/C + T/T	0.903 (0.394–2.072)	0.810	
Recessive	C/C + C/T	1		147.484
	T/T	0.625 (0.115–3.298)	0.586	
Overdominant	T/T + C/C	1		147.762
	T/C	1.001 (0.424–2.362)	0.998	
Additive	—	0.870 (0.441–1.717)	0.688	147.603
Females				
Codominant	CC	1		271.981
	CT	0.528 (0.284–0.981)	0.043	
	TT	1.033 (0.278–3.837)	0.961	
Dominant	CC	1		269.092
	T/C + T/T	0.579 (0.318–1.053)	0.073	
Recessive	C/C + C/T	1		272.068
	T/T	1.368 (0.382–4.907)	0.630	
Overdominant	T/T + C/C	1		267.927
	T/C	0.526 (0.288–0.961)	0.037	
Additive	—	0.748 (0.471–1.187)	0.218	270.822

**Table 6:** Frequency of *MMP-2 (-1306 C/T)* genotype in the patients with pituitary adenoma (PA) and in the control group by PA invasiveness.

Gene marker	Genotype/allele	Frequency (%)					
		Control group *n* (%) (*n* = 318)	*p* HWE	Noninvasive PA group *n* (%) (*n* = 37)	*p* HWE	Invasive PA group *n* (%) (*n* = 47)	*p* HWE
*MMP-2 (-1306) Rs243865*	Genotype		0.383		0.064		0.5823
	C/C	190^*∗*^ (59.75)		13^*∗*^ (35.1)		29 (61.7)	
	C/T	108^*∗∗*^ (33.96)		22^*∗∗*^ (59.5)		15 (31.9)	
	T/T	20 (6.29)		2 (5.4)		3 (6.4)	
	Total	318 (100)		37 (100)		47 (100)	
	Allele						
	C	488 (76.72)		48 (64.86)		73 (77.66)	
	T	148 (23.33)		26 (35.14)		21 (22.34)	

^**∗**^ *p* = 0.0049.

^**∗∗**^ *p* = 0.0035.

MMP: matrix metalloproteinase; *p* value: significance level (alfa = 0.05); *p*-value HWE: significance level (alfa = 0.05) by Hardy-Weinberg equilibrium.

**Table 7:** Frequency of *MMP-2 (-1306 C/T)* genotype in the patients with pituitary adenoma (PA) and in the control group by PA recurrences.

Gene marker	Genotype/allele	Frequency (%)					
		Control group *n* (%) (*n* = 318)	*p* HWE	Nonrecurrence PA group *n* (%) (*n* = 71)	*p* HWE	Recurrence PA group *n* (%) (*n* = 13)	*p* HWE
*MMP-2 (-1306) Rs243865*	Genotype		0.383		0.3958		0.5121
	C/C	190^*∗*^ (59.75)		33^*∗*^ (46.5)		9 (69.2)	
	C/T	108 (33.96)		33 (46.5)		4 (30.8)	
	T/T	20 (6.29)		5 (7.0)		0 (0)	
	Total	318 (100)		71 (100)		13 (100)	
	Allele						
	C	488 (76.72)		99 (69.72)		22 (84.62)	
	T	148 (23.33)		43 (30.28)		4 (15.38)	

^**∗**^ *p* = 0.0468.

MMP: matrix metalloproteinase; *p* value: significance level (alfa = 0.05); *p*-value HWE: significance level (alfa = 0.05) by Hardy-Weinberg equilibrium.

**Table 8:** Binomial logistic regression analysis in noninvasive and nonrecurrence pituitary adenoma (PA) and in the control group.

Model	Genotype	OR (CI 95%)	*p* value	AIC
Noninvasive				
Codominant	CC	1		234.221
	CT	2.977 (1.442–6.148)	**0.003**	
	TT	1.462 (0.308–6.944)	0.633	
Dominant	CC	1		233.197
	T/C + T/T	2.740 (1.346–5.581)	**0.005**	
Recessive	C/C + C/T	1		241.285
	T/T	0.851 (0.191–3.797)	0.833	
Overdominant	T/T + C/C	1		232.431
	T/C	2.852 (1.422–5.721)	**0.003**	
Additive	—	1.782 (1.065–2.982)	**0.028**	236.667
Nonrecurrence				
Codominant	CC	1		371.421
	CT	1.759 (1.028–3.011)	**0.039**	
	TT	1.439 (0.505–4.102)	0.496	
Dominant	CC	1		369.564
	T/C + T/T	1.709 (1.019–2.868)	**0.042**	
Recessive	C/C + C/T	1		373.646
	T/T	1.129 (0.409–3.117)	0.815	
Overdominant	T/T + C/C	1		369.859
	T/C	1.689 (1.003–2.843)	**0.049**	
Additive	—	1.423 (0.953–2.123)	0.084	370.788
